# Unusual Suspect: Streptococcus pyogenes as a Cause of Pneumonia

**DOI:** 10.7759/cureus.42495

**Published:** 2023-07-26

**Authors:** Lisandra Nunez Cuello, Kavisha Jain, Loren Inigo-Santiago

**Affiliations:** 1 Department of Internal Medicine, Danbury Hospital, Danbury, USA; 2 Department of Pulmonary and Critical Care Medicine, Danbury Hospital, Danbury, USA

**Keywords:** severe community-acquired pneumonia, chest tube, spontaneous empyema, septic shock [ss], streptococcus pyogenes infection

## Abstract

A 73-year-old male patient with a history of hypertension and coronary artery disease presented to the hospital with dyspnea, nonproductive cough, sore throat, and fever. Prior to presentation, the patient was treated for over a week for upper respiratory infection with conservative management. Images were positive for extensive pleural effusions and consolidations, particularly in the right lung. The patient was admitted with the diagnosis of septic shock secondary to acute hypoxic respiratory failure secondary to community-acquired multifocal pneumonia. Blood and pleural fluid cultures confirmed the diagnosis of *Streptococcus pyogenes* pneumonia complicated with empyema. Despite a challenging hospital course, including renal failure requiring dialysis and surgical interventions for empyema, the patient improved after completing a 21-day antibiotic regimen. Invasive Group A *Streptococcus* (iGAS) infections can range from mild to life-threatening. Certain viral infections, such as influenza, can exacerbate these infections, particularly in vulnerable populations like the elderly or those with chronic illnesses. Treatment predominantly involves beta-lactams, supplemented by clindamycin in septic cases.

## Introduction

*Streptococcus pyogenes*, or Group A *Streptococcus* (GAS), while more commonly associated with mild infections, can occasionally cause severe conditions such as acute pneumonia [1]. Historically, the virulence and complexity of GAS infections have been linked with viral outbreaks like varicella and influenza [2].^ ^Despite being uncommon, *Streptococcus pyogenes* pneumonia (SPP) often requires intricate critical care management strategies due to its high mortality rate.

In this report, we present the case of a patient who developed SPP, initially presenting with common symptoms of an upper respiratory infection. This quickly escalated into a severe disease, requiring intensive medical interventions. The diagnosis of SPP was confirmed upon the detection of *Streptococcus pyogenes* in both blood culture and pleural fluid analysis. 

## Case presentation

A 73-year-old male presented to the emergency department (ED) with chief complaints of dyspnea, nonproductive cough, sore throat, and fever. His symptoms had developed over the course of a week and three days prior to admission, he visited his primary care doctor, who performed Influenza and coronavirus disease 2019 (COVID-19) tests, both of which came back negative. Consequently, the patient was managed conservatively. His past medical history included hypertension and coronary artery disease. He was a non-smoker and denied alcohol consumption.

On presentation, his temperature was 36 °C, heart rate 114 beats per minute, respiratory rate 32 breaths per minute, blood pressure 100/60 mmHg, and oxygen saturation was 83% initially on 6 L of oxygen. Upon physical examination, he was dyspneic, the oral mucosa showed no erythema, and the lung examination revealed crackles throughout the right lung. The laboratory blood work indicated that the electrolytes were within normal limits; the remaining pertinent work-up is summarized in Table 1. Additionally, arterial blood gas analysis was performed (Table 2).

**Table 1:** Initial blood work MRSA: methicillin-resistant *Staphylococcus aureus; *PCR: polymerase chain reaction

Lab investigation	Results	Reference range
White blood cell (WBC) count	35.7 x10^9^/L	3.5-10
Hemoglobin	14.2 g/dL	13.5-17
Platelet count	420 x10^9^/L	150-400
Creatinine (cr)	3.43 mg/dL	0.67-1.23
Lactic acid	9.2 mmol/L	<1.9
MRSA PCR nasal swab	Negative	

**Table 2:** Arterial blood gases pCO2: partial pressure of carbon dioxide,  HCO3: bicarbonate, pO2: partial pressure of oxygen

Arterial Blood Gases	Results	Reference range
pH	7.39	7.31-7.42
pCO2	25 mmHg	35-48
HCO3	15 mmol/L	22-26
pO2	52 mmHg	>70
O2 Arterial Saturation	87 %	>95

**Figure 1:**
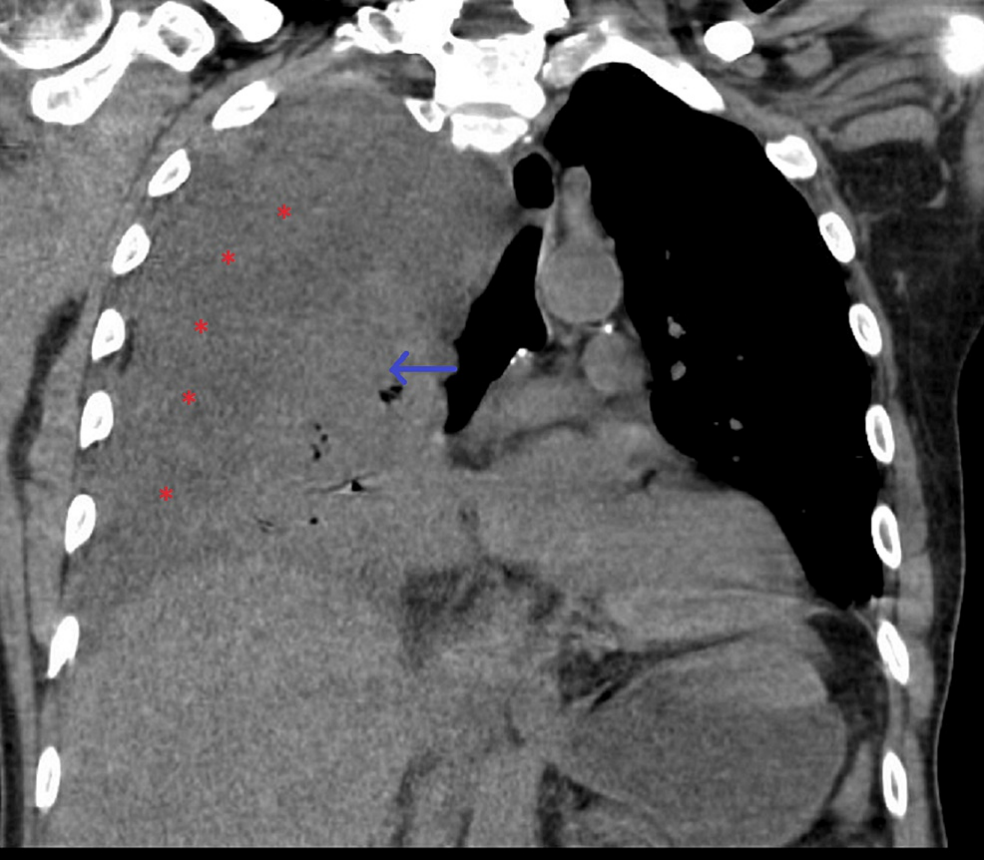
CT of the chest without contrast, coronal cuts, showed large right pleural effusion (red asterisk) with extensive atelectasis and consolidation of the right lung (blue arrow).

A chest X-ray demonstrated multifocal areas of airspace with a small right pleural effusion. The computed tomography (CT) of the chest revealed extensive right pleural effusion (Figure 1) with extensive atelectasis and consolidation involving the right upper lobe, right middle lobe, and right lower lobe, as well as nonspecific patchy lingular and left lower lobe opacity. 

In the ED, the patient was started on fluid resuscitation, and due to persistent hypotension, he was started on vasopressors. He continued to deteriorate and had persistent hypoxemia. The decision was made to intubate the patient emergently. The initial antibiotic regimen was ceftriaxone and metronidazole. He was admitted with the working diagnosis of septic shock secondary to acute hypoxic respiratory failure in the setting of community-acquired multifocal pneumonia. Given the right-sided pleural effusion, a chest tube was placed. This drained purulent material and pleural fluid analysis indicated empyema with the following results: lactate dehydrogenase (LDH) >2500 U/L, pH <6.80. The WBC count was 140,343/cumm with a differential of 81% polymorphonuclears. Cytology was negative for malignant cells. Upon obtaining blood culture and pleural fluid results consistent with *Streptococcus pyogenes*, clindamycin was added due to possible streptococcal toxic shock syndrome.

**Figure 2:**
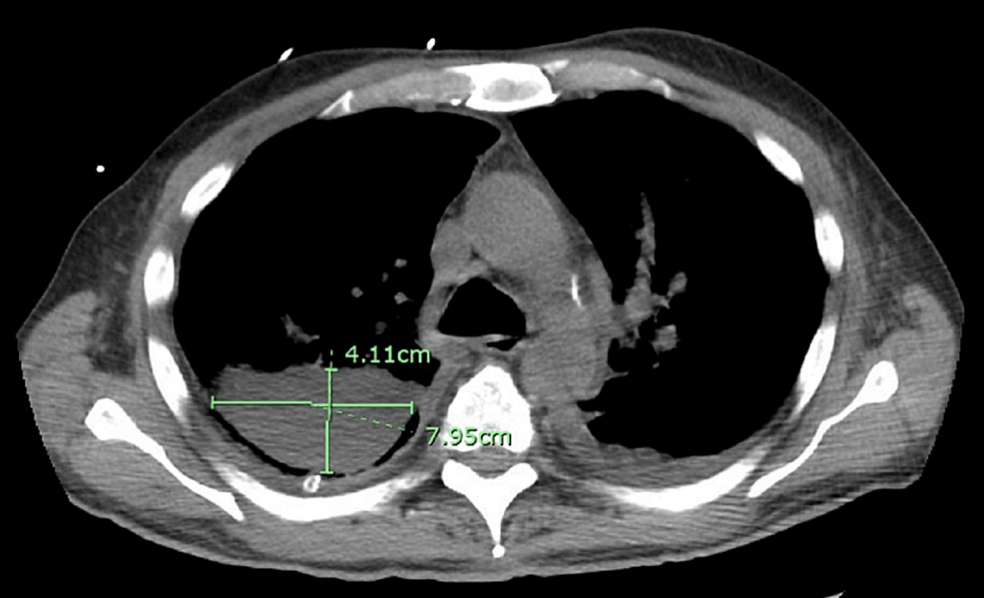
CT chest without contrast, axial cuts, showed right lung parenchyma with loculated effusion along the major fissure measuring 8.0 x 4.1 cm.

**Figure 3:**
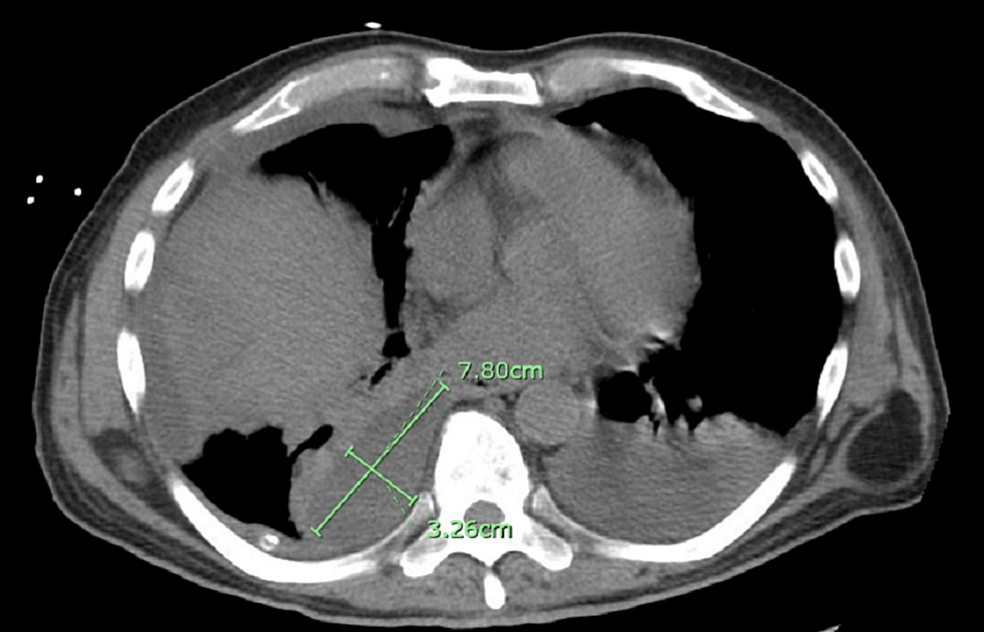
CT chest without contrast, axial cuts, showed right lung parenchyma with medial right lower loculated pleural effusion measures approximately 7.8 x 3.3 cm.

A repeated CT scan of the chest obtained on the fourth day of hospitalization demonstrated an interval increase in the size of the right pleural effusion, new loculated effusions in the right hemithorax (Figure 2, 3), and a new moderate left pleural effusion, as well as new ground-glass opacities in the left upper lobe and patchy ground-glass opacities in the right upper lobe. Cardiothoracic surgery was consulted for possible video-assisted thoracoscopic surgery (VATS), but due to the inability to ventilate a single lung in the setting of severe pneumonia, VATS could not be performed. A right-sided thoracotomy was performed with fibrinous material evacuation and loculation dissection. During the course of his hospitalization, the patient also developed renal failure, which required hemodialysis. After a prolonged hospital course and completing 21 days of antibiotic treatment, the patient was discharged.

## Discussion

GAS, or *Streptococcus pyogenes*, is a Gram-positive bacteria known to cause asymptomatic colonization and a wide array of infections ranging from impetigo and pharyngitis to post-streptococcal immunological sequelae, such as acute rheumatic fever and acute glomerulonephritis [1,3]. Life-threatening invasive GAS (iGAS) is defined as the isolation of pathogens from sterile sites, the most notable examples are necrotizing fasciitis, SPP, streptococcal myositis, and streptococcal toxic shock syndrome (TSS) [2,4].

In the previous century, large outbreaks were observed as an association between GAS and viral infections, notably measles and the Spanish influenza pandemic [2,5]. More recently, the swine flu (H1N1) virus pandemic had a similar impact [6]. The annual incidence of iGAS ranges from 1.5 to 8.1 cases per 100,000 persons [3,7]. Pneumonia is a common manifestation of iGAS, noted in 10-16% of cases [2,8,9] with a mortality rate of 17% in more recent studies [2]. 

Patients who are particularly susceptible include those over 65 years of age, notably patients with underlying conditions such as type 2 diabetes mellitus, cancer, alcohol use disorder, and chronic obstructive pulmonary disease [10,11]. Most cases have been reported during winter and spring, exhibiting a significant correlation with viral infections such as influenza, respiratory syncytial virus (RSV), and metapneumovirus [2]. The exact pathogenesis of GAS infection in patients with influenza is not yet fully understood. It is suggested that the influenza infection might increase fibronectin production, leading to GAS binding, respiratory damage, and immunosuppression, promoting GAS invasion [6,12]. Interestingly, in a study with a healthy military population, influenza immunization indicated potential protective effects against GAS disease. However, due to study limitations, these results should be viewed with caution [13]. 

The M protein gene (emm) encodes for the principal pathogenic factor of GAS, the M protein. It is the hypervariability of this gene that allows for the identification of the strains, with the most virulent being M-type 1 and M-type 3, which are associated with increased mortality [8,14]. The M protein interferes with both complement and antibody activation, reducing phagocytosis and promoting the formation of microcolonies, which leads to rapid multiplication in the blood [4,14]. Additionally, streptococcal pyrogenic exotoxin A, a superantigen, can stimulate T cells by bypassing the usual antigen processing and presentation, leading to excessive inflammatory response [4]. 

Patients often present with a rapid onset of dyspnea, fever, and chest pain. The most distinctive characteristic is the rapid accumulation of pleural effusion, observed in approximately 80% of patients with SPP compared to other causes of pneumonia [1]. In addition, these patients may also develop empyema, bacteremia, and necrotizing pneumonia, with the majority being admitted to the ICU due to the severity of the disease [2,15]. 

Beta-lactams are the preferred treatment for both complicated and uncomplicated infections. Clindamycin is recommended as adjunctive therapy for patients presenting with sepsis and should never be used as a monotherapy [16]. The advantages include its direct effects on enhancing phagocytosis, increasing tissue penetration, and decreasing bacterial toxin production.

## Conclusions

This case report highlights the severity of GAS pneumonia, accompanied by complications like empyema and septic shock. Early recognition, appropriate antibiotic therapy, and timely intervention are crucial in managing these cases. The role of adjunctive therapies, such as clindamycin, should be considered in severe presentations. 

## References

[1] Birch C, Gowardman J (2000). Streptoccocus pyogenes: a forgotten cause of severe community-acquired pneumonia. Anaesth Intensive Care.

[2] Wilson PA, Varadhan H (2020). Severe community-acquired pneumonia due to Streptococcus pyogenes in the Newcastle area. Commun Dis Intell (2018).

[3] Herrera AL, Huber VC, Chaussee MS (2016). The association between invasive group a streptococcal diseases and viral respiratory tract infections. Front Microbiol.

[4] Steer AC, Lamagni T, Curtis N, Carapetis JR (2012). Invasive group a streptococcal disease: epidemiology, pathogenesis and management. Drugs.

[5] Morens DM, Taubenberger JK (2015). A forgotten epidemic that changed medicine: measles in the US Army, 1917-18. Lancet Infect Dis.

[6] Jean C, Louie JK, Glaser CA (2010). Invasive group A streptococcal infection concurrent with 2009 H1N1 influenza. Clin Infect Dis.

[7] Stockmann C, Ampofo K, Hersh AL (2012). Evolving epidemiologic characteristics of invasive group A streptococcal disease in Utah, 2002-2010. Clin Infect Dis.

[8] Nelson GE, Pondo T, Toews KA (2016). Epidemiology of invasive group A streptococcal infections in the United States, 2005-2012. Clin Infect Dis.

[9] Muller MP, Low DE, Green KA, Simor AE, Loeb M, Gregson D, McGeer A (2003). Clinical and epidemiologic features of group a streptococcal pneumonia in Ontario, Canada. Arch Intern Med.

[10] Siegel MD, Kimmel R (2006). A 48-year-old woman with pneumonia, shock, and a rash. Chest.

[11] Tamayo E, Montes M, Vicente D, Pérez-Trallero E (2016). Streptococcus pyogenes pneumonia in adults: clinical presentation and molecular characterization of isolates 2006-2015. PLoS One.

[12] Akuzawa N, Kurabayashi M (2016). Bacterial pneumonia caused by Streptococcus pyogenes infection: a case report and review of the literature. J Clin Med Res.

[13] Lee SE, Eick A, Bloom MS, Brundage JF (2008). Influenza immunization and subsequent diagnoses of group A streptococcus-illnesses among U.S. Army trainees, 2002-2006. Vaccine.

[14] Santagati M, Spanu T, Scillato M (2014). Rapidly fatal hemorrhagic pneumonia and group A Streptococcus serotype M1. Emerg Infect Dis.

[15] Barnham M, Weightman N, Anderson A, Pagan F, Chapman S (1999). Review of 17 cases of pneumonia caused by Streptococcus pyogenes. Eur J Clin Microbiol Infect Dis.

[16] Lecronier M, Elabbadi A, Mekontso Dessap A, de Prost N (2017). Short and long-term outcomes of Streptococcus pyogenes pneumonia managed in the intensive care unit. Infect Dis (Lond).

